# Management and outcomes of paediatric achalasia: multicentre retrospective study in the UK

**DOI:** 10.1093/bjsopen/zraf139

**Published:** 2026-01-20

**Authors:** Jonathan J Neville, Esther Westwood, Amanda Ladell, George S Bethell, Rachel Harwood, Nigel J Hall, the PSTRN Collaborators, Mohamad E Abdullah, Paul Charlesworth, Liza Y W Chong, Claire Clark, David Colvin, Ahish Desai, Hany Gabra, Stefano Giuliani, Lucinda Tullie, Dale Gracie, Naotunna Palliyage Nuwanthika Karunaratne, Saravanakumar Paramalingam, Chiara Pelosi, Shazia Perveen, Amulya Saxena, Giampiero Soccorso, Chloe Roy, Iain Yardley, Dylan Wills, Rachel Tan Wei Ying

**Affiliations:** Great Ormond Street Institute of Child Health, University College London, London, UK; Department of Paediatric Surgery, Evelina Children’s Hospital, London, UK; Achalasia Action, London, UK; University Surgery Unit, University of Southampton, Southampton, UK; Department of Paediatric Surgery, Alder Hey Children’s Hospital, Liverpool, UK; University Surgery Unit, University of Southampton, Southampton, UK

**Keywords:** paediatrics, child health, achalasia, surgery, gastroenterology

## Abstract

**Background:**

Achalasia is rare disease in children and young people (CYP) that causes significant symptoms and often requires invasive interventions. There is currently no consensus on the optimal management strategy. This study investigated the current management and outcomes of CYP with achalasia in the UK.

**Methods:**

A retrospective study was conducted of CYP (aged ≤ 16 years) diagnosed with achalasia between 2011 and 2021 in the UK. The study was co-designed with the patient group Achalasia Action. Data were collected from patient records. The primary outcome was treatment success.

**Results:**

In all, 126 patients were included from 13 UK centres; 64 of the patients (50.8%) were male and the median age at diagnosis was 12 (interquartile range (i.q.r.) 9–14) years. The most frequent presenting features were dysphagia (73.8%), vomiting (53.2%), and weight loss (38.9%). The median time from symptom onset to diagnosis was 11 (i.q.r. 6–24) months. Treatment success was achieved in 55 of 120 patients (45.8%) after first-line intervention. Heller’s cardiomyotomy (HCM) as the first-line intervention had a higher success rate than endoscopic balloon dilatation (EBD; (52 of 72 (72%) *versus* 3 of 48 (6%), respectively; *P* < 0.001). However, overall HCM had a higher frequency of complications than EBD (17 of 98 (17%) *versus* 3 of 57 (5%), respectively; *P* = 0.045). In the entire cohort, 53% of patients reported symptoms at the 1-year follow-up.

**Conclusions:**

Variation exists in the management of CYP with achalasia in the UK. The highest rates of treatment success were associated with HCM. Many CYP remain symptomatic after treatment and require multiple interventions. The present data can be used to inform management decisions in CYP with achalasia.

## Teaser

Achalasia is rare disease in children and young people (CYP) that causes significant symptoms and often requires invasive interventions. This retrospective study of 126 CYP with achalasia diagnosed between 2011 and 2021 at 13 centres in the UK found variation in the management of CYP with achalasia. The highest rates of treatment success were associated with Heller’s cardiomyotomy, but many CYP remain symptomatic after treatment and require multiple interventions.

## Introduction

Achalasia is a rare disease of oesophageal dysmotility with an estimated incidence in children and young people (CYP) of 0.11–1.80 per 100 000 per year^1,2^. Achalasia is characterized by disordered peristalsis of the oesophageal body, high resting tone of the lower oesophageal sphincter (LOS), and a failure of the LOS to relax on swallowing. Patients develop progressive dysphagia, vomiting, and weight loss. Diagnosis of achalasia is often delayed in CYP due to the rarity of the disease, and achalasia can be misdiagnosed as a more common condition with similar presenting features^3^.

Medical and surgical treatment strategies aim to facilitate swallowing and improve symptoms by reducing LOS pressure^2^. Endoscopic therapies include botulinum toxin (Botox) injection into the LOS, endoscopic balloon dilatation (EBD), and peroral endoscopic myotomy (POEM). Surgical treatment comprises Heller’s cardiomyotomy (HCM), with or without concomitant fundoplication. However, there is no clear consensus on the optimal management strategy for CYP with achalasia. Compared with EBD, HCM is associated with a higher rate of clinical improvement but an increased rate of procedural complications^3,4^. Studies have reported that a large proportion of CYP remain symptomatic for long periods, even after both endoscopic and surgical management^3,5^. Early reports of POEM in CYP show a good response to treatment that is similar to that in adult patients, but long-term outcome data are lacking^6–9^. Due to the rarity of achalasia in CYP, the current literature is based on small, single-centre retrospective studies and, to date, there have been no randomized clinical trials comparing different treatment strategies in this cohort. Moreover, data from interventional studies in adult achalasia patients may not translate to CYP due to differences in anatomy, physiology, clinical features, and the requirement for long follow-up periods.

The aim of the present study was to describe the clinical presentation, management, and outcomes of CYP diagnosed with achalasia in the UK. A secondary objective was to describe the success of first-line interventions.

## Methods

This retrospective multicentre study was conducted at specialist paediatric surgery centres in the UK. CYP (aged ≤ 16 years) diagnosed with achalasia between 1 January 2011 and 31 December 2021 were eligible for inclusion. In order to capture the complete scope of practice in the UK, criteria for a diagnosis of achalasia were not specified. As such, a diagnosis of achalasia was made based on the presenting features of each patient and the results of investigations undertaken at each centre.

This study was co-designed with the patient group Achalasia Action. During the development of the study protocol, a focus group was held with five parents of CYP with achalasia, and one adult with achalasia diagnosed in childhood. The study protocol was revised based on the focus group’s feedback. Feedback was sought from a further six patient representatives via an online survey. The patient group was specifically interested in understanding whether a particular first-line treatment strategy for CYP with achalasia was superior, and to what extent dieticians and clinical psychologists were involved in patient care.

Data were extracted from electronic patient records by the local team and uploaded to a secure online REDCap database^10^. Data regarding patient demographics, co-morbidities, clinical features, investigations, interventions, disease and procedural outcomes, and outcomes at follow-up were collected. Local approvals were obtained at each participating centre.

The primary outcome was treatment success, defined as symptom improvement (any degree of improvement of patient-reported symptoms), combined with the requirement for no further treatments, at the time of most recent follow-up. Currently, there are no validated measures of treatment success in CYP with achalasia^11^. This primary outcome was selected because it is measurable with retrospective data and has been used in previous multicentre studies of achalasia in CYP^4,12,13^. Therefore, patients with treatment success may still report symptoms. Other outcomes reported included complications of interventions, the requirement for repeat interventions, the success of medical management, growth, postintervention incidence of patient-reported reflux and dysphagia symptoms, and symptomatology at the 1- and 5-year follow-up. The presence or absence of patient-reported symptoms were collected from patient records. EBD was pneumatic or hydrostatic depending on clinician experience and preference. Additional procedures, such as Botox injection into the LOS, were performed at the discretion of the attending clinician. Performance of multiple EBD within a 3-month period was classified as a single course of treatment. Surgical techniques for HCM and fundoplication were not standardized but consisted of open, laparoscopic, or robotic HCM with variable-length oesophageal and cardiac myotomies^14^. Performance of a fundoplication and the choice of fundoplication type were based on surgeon preference^14^. Achalasia subtype on manometry, as per the Chicago classification^15^, was extracted from formal manometry reports where available. Weight Z-score was calculated using sex-specific Centers for Disease Control growth charts based on age^16^.

Data are summarized as the median with interquartile range (i.q.r.) unless otherwise specified. Continuous non-parametric data were compared using the Mann–Whitney *U* (unpaired) or Wilcoxon signed-rank (paired) tests. Categorical data were compared using χ^2^ or Fisher’s exact tests as appropriate. Time-to-event curves were compared using the log-rank test. Data analysis was conducted in SPSS^®^ v29.0 (IBM, Armonk, NY, USA) and GraphPad Prism v10 (GraphPad Software, San Diego, CA, USA). All tests were two-sided and *P* < 0.05 was considered significant.

## Results

In all, 126 CYP from 13 UK centres were included in the study. The median age at diagnosis was 11.9 (i.q.r. 9.4–13.5) years and 64 CYP (50.8%) were male. Ethnicity was reported for 100 patients; of these 100 patients, 67 (67.0%) were White, 19 (19.0%) were Asian or Asian British, 10 (10.0%) were Black, Black British, Caribbean or African, and 4 (4.0%) were mixed or multiple ethnic groups. Across the entire study cohort, a relevant past medical history was reported for 23 CYP (18.3%), with 5 CYP (4.0%) having a diagnosis of Allgrove syndrome, 6 (4.8%) having a diagnosis of asthma, 4 (3.2%) having a diagnosis of intestinal pseudo-obstruction, and 1 (0.8%) having chronic constipation.

The most common presenting features were dysphagia (73.8%), vomiting (53.2%), and weight loss (38.9%; *Table S1*). The median time from the onset of clinical features to diagnosis was 11 (i.q.r. 6–24) months. The Eckardt score was reported at diagnosis for 26 of 126 CYP (20.6%), with the median score being 5.5 (i.q.r. 3–6.8).

Across the entire study cohort, there was evidence of an initial misdiagnosis in 28 CYP (22.2%), most frequently resulting in an erroneous diagnosis of gastro-oesophageal reflux disease (16 CYP, 12.7%). Four CYP (3.2%) were diagnosed with eosinophilic oesophagitis. Other diagnoses that preceded an achalasia diagnosis were cow’s milk protein allergy (1), behavioural vomiting (1), infection (1), feeding difficulties without a formal diagnosis (4), and recurrent chest infections (1).

Twenty-three CYP (18.3%) required alternative feeding strategies before treatment, including nasogastric tube feeding in 18 (14.3%), nasojejunal tube feeding in 1 (0.8%), gastrostomy feeding in 3 (2.4%), and parental nutrition in 1 (0.8%).

A barium swallow was performed in 115 CYP (91.3%), with radiographic findings suggesting achalasia in 99 of these 115 CYP (86.1%). An oesophagogastroduodenoscopy was performed in 78 of 126 CYP (61.9%). Manometry was undertaken in 81 of 126 CYP (64.3%), and was conventional in 22 (27%) and high-resolution in 59 (73%). Chicago classification subtype was reported for 53 of 81 CYP (65%), but was not associated with clinical features, first-line treatment success, or the presence of symptoms at the 1-year follow-up (*Table 1*).

**Table 1:** Chicago classification achalasia subtypes, clinical features and outcomes in patients undergoing manometry

	All patients (*n* = 81)	Achalasia subtype				P*
		Unclassified (*n* = 28)	Type I (*n* = 8)	Type II (*n* = 41)	Type III (*n* = 4)	
**Clinical features**						
Dysphagia	46 (57%)	17 (61%)	7 (88%)	35 (85%)	4 (100%)	0.910
Regurgitation	32 (40%)	11 (39%)	2 (25%)	15 (37%)	4 (100%)	0.013†
Vomiting	39 (48%)	10 (36%)	6 (75%)	21 (51%)	2 (50%)	0.341
Chest pain	32 (40%)	6 (21%)	1 (13%)	6 (14%)	0 (0%)	0.671
Failure to gain weight	4 (5%)	1 (4%)	1 (13%)	2 (5%)	0 (0%)	0.568
Weight loss	35 (43%)	8 (29%)	4 (50%)	21 (51%)	2 (50%)	0.927
Reflux	8 (10%)	3 (11%)	2 (25%)	3 (7%)	0 (0%)	0.199
Nocturnal cough	8 (10%)	5 (18%)	1 (13%)	2 (5%)	0 (0%)	0.568
First-line treatment success	41 (51%)	14 (50%)	3 (38%)	20 (49%)	4 (100%)	0.105
Symptoms at 1-year follow-up (*n* = 57)	32 of 57 (56%)	9 of 18 (50%)	1 of 4 (25%)	21 of 32 (66%)	1 of 3 (33%)	0.191

Values are *n* (%) unless otherwise stated. †After post hoc pairwise comparison, the relationship between regurgitation and achalasia subtype becomes non-significant. *χ^2^ or Fisher’s exact test.

Of the 126 CYP in this study, 20 (15.9%) were discussed in a multidisciplinary team meeting. Decisions regarding treatment for the CYP were made predominantly by paediatric surgeons (105, 83.3%) and gastroenterologists (72, 57.1%). Adult surgeons or gastroenterologists were involved in decisions for 9 CYP (7.1%). There was dietician involvement for 55 of 126 CYP (43.7%) and clinical psychologist input for 7 of 126 CYP (5.6%).

Medical treatments in the form of prokinetics, calcium channel blockers, and proton pump inhibitors were used in 53 of 126 CYP (42.1%). The duration of treatment was reported for 43 of 53 CYP (81%) and was a median of 12 (i.q.r. 6–13.5) months. Of the 47 patients receiving proton pump inhibitors, 14 (30%) reported some symptomatic improvement. Otherwise, medical treatments were reported as ineffective.

First-line interventions across the entire cohort were EBD in 48 CYP (38.1%; including three patients who received EBD and Botox) and HCM in 72 CYP (57.1%). Six patients received no intervention. No POEM procedures were reported. Median time from diagnosis to first-line treatment was 2 months (0–5). Treatment success was reported for 55 of 120 patients (45.8%) after first-line treatment (*Table 2*). Both age and weight at diagnosis were significantly higher among CYP with treatment success than among those with treatment failure (median age 12.5 (i.q.r. 11.3–14.0) *versus* 10.8 (i.q.r. 8.1–12.4) years, respectively (*P* = 0.006); weight at diagnosis 40 (i.q.r. 31–47) *versus* 31.0 (i.q.r. 21.5–43.8) kg, respectively (*P* = 0.009)). Treatment success was also significantly higher in CYP managed with HCM compared to EBD (52 of 72 (72%) *versus* 3 of 48 (6%), respectively; *P* < 0.001). After successful first-line treatment, symptom improvement was maintained for a median of 12 (i.q.r. 4–27) months. Undertaking multiple EBD within 3 months, compared with a single EBD, was not associated with an increased rate of treatment success (*P* = 0.227).

**Table 2:** Comparison of outcomes based on first-line treatment modality

	HCM (*n* = 72)	EBD (*n* = 48)	P*
Treatment success	52 (72%)	3 (6%)	< 0.001
Any complications	8 (11%)	1 (2%)	0.137
Symptomatic at 1 year (*n* = 86†)	30 of 56 (54%)	16 of 30 (53%)	0.983

Values are *n* (%) unless otherwise stated. †Of 88 patients with 1-year follow-up data, 86 had undergone at least one intervention. HCM, Heller’s cardiomyotomy; EBD, endoscopic balloon dilatation. *χ^2^ or Fisher’s exact test.

**Fig. 1:**
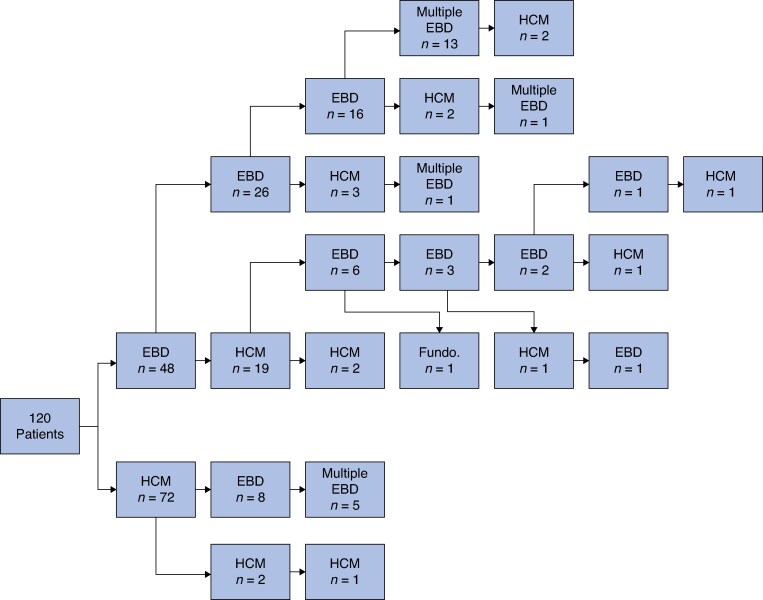
Flow chart showing serial EBD, HCM, or fundoplication EBD, endoscopic balloon dilatation; HCM, Heller’s cardiomyotomy; Fundo., fundoplication.

**Fig. 2:**
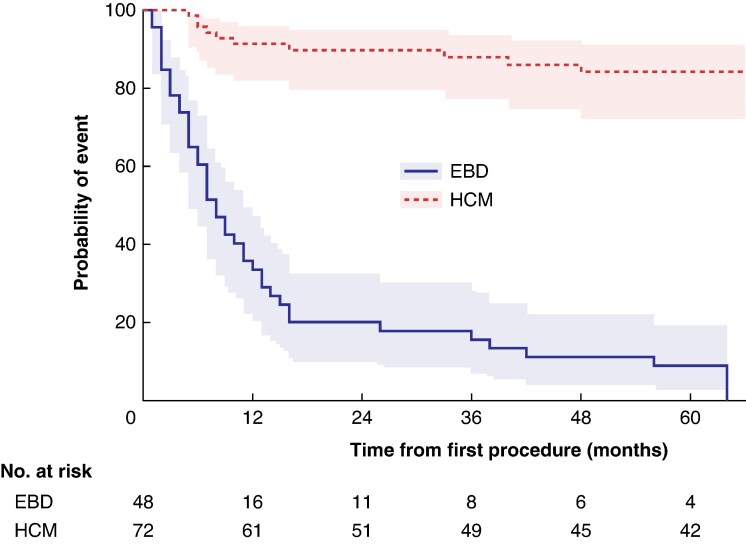
Time to second procedure in children and young people managed with first-line EBD or HCM Shaded areas indicate 95% confidence intervals. EBD, endoscopic balloon dilatation; HCM, Heller’s cardiomyotomy.

Following first-line treatment, 55 of 120 CYP (45.8%) required at least one subsequent intervention (*Fig. 1*). The time to second intervention was longer in CYP managed with first-line HCM than among those managed with EBD (log-rank hazard ratio 11.1; 95% confidence interval 6.0 to 20.6; *P* < 0.001; *Fig. 2*).

Complications were more frequently experienced by CYP undergoing HCM than EBD (17 of 98 (17%) *versus* 3 of 57 (5%), respectively; *P* = 0.045). The surgical complications among the 98 patients undergoing HCM were oesophageal or gastric perforations (9, 9%), reflux (3, 3%), incomplete myotomy (2, 2%), readmission with pain or wound complications (2, 2%), and development of a pleural effusion (1, 1%). One patient with an oesophageal perforation required admission to paediatric intensive care. The speciality of the surgeon performing the HCM did not affect the complication rate or the requirement for a redo HCM (*Table S2*). Among the 57 patients undergoing EBD, complications included aspiration (2, 4%) and bleeding at the gastro-oesophageal junction (1, 2%).

Among the 98 CYP undergoing surgery, the first HCM was laparoscopic in 88 (90%), laparoscopic converted to open in 3 (3%), robotic in 4 (4%), and open in 3 (3%). Of the seven redo HCMs, 6 (86%) were laparoscopic and 1 (14%) was open. One patient underwent a second redo HCM, which was a laparoscopic converted to open procedure.

At HCM, fundoplication was performed in 66 of 98 CYP (67%). Among the 63 patients (95%) with reported data, the wrap was anterior 180° in 32 (51%), anterior 200° in 21 (33%), anterior 270° in 5 (8%), and posterior 270° in 2 (3%). The intraoperative complication rate, treatment success, and the requirement for a redo HCM were similar between HCMs performed with and without a fundoplication (*Table S3*). At 1 year, symptomatic dysphagia was more frequent in those who underwent fundoplication (32% *versus* 13%; *P* = 0.049), whereas gastro-oesophageal reflux disease symptoms were similar between HCM performed with and without a fundoplication (9% *versus* 8%, respectively; *P* = 0.714). Only one patient underwent a laparoscopic fundoplication after HCM at 53 months.

An endoscopic procedure before HCM did not significantly increase the rate of surgical complications (10 of 71 (14%) treatment-naïve HCM *versus* 7 of 27 (26%) prior EBD; *P* = 0.233). However, the rate of requiring a subsequent intervention after initial HCM surgery was significantly higher in CYP who had undergone a prior EBD (11 of 71 (15%) treatment-naïve HCM *versus* 12 of 27 (44%) prior EBD; *P* = 0.003).

Follow-up data were available for 88 of 126 patients (69.8%) at 1 year. Symptoms were reported by 47 of these 88 patients (53%) and were most commonly dysphagia (27, 57%), regurgitation (10, 21%), and reflux (10, 21%; *Table 2*). Morbidities were reported for 15 of 88 CYP (17%) with follow-up data, and included gastro-oesophageal reflux (9, 60%), oesophagitis (3, 20%), and oesophageal strictures (3, 20%). Five-year follow-up data were available for 22 of 126 patients (17.5%), with 16 of these patients (73%) reporting symptoms. The median weight-for-age Z score had increased from diagnosis to the 1-year follow-up (−0.55 (i.q.r. −1.38–0.18) *versus* 0.60 (i.q.r. −0.51–1.26); *P* < 0.001; *Fig. S1*). Forty-five CYP (35.7%) in the cohort had transitioned to adult services.

## Discussion

This study reports on the management and outcomes of CYP with achalasia from 13 specialist paediatric centres in the UK. The key findings of the study were variations in both diagnostic and management approaches, and that over half of all CYP with achalasia remained symptomatic 1 year after treatment. Of the treatments provided, HCM was associated with a significantly higher rate of treatment success and a longer time to second-line treatment *versus* EBD, but HCM also carried a higher rate of complications.

One-fifth of the CYP were initially misdiagnosed, and the median time from symptom onset to diagnosis was 11 months. Smits *et al.*^3^ observed a 15% misdiagnosis rate and similarly reported eosinophilic oesophagitis and gastro-oesophageal reflux as the most common misdiagnoses. Other studies have reported the time from symptom onset to diagnosis as being around 1 year^3,4,17^. This highlights the need for a high index of suspicion and early manometry to identify achalasia in CYP.

In adult patients with achalasia, the Chicago classification predicts outcomes following interventional procedures, such that patients with type I and III achalasia tend to benefit from HCM or POEM as opposed to EBD^18,19^. Similar to previous reports in CYP^4^, type II achalasia was most prevalent in the present cohort. Manometric subtype was not associated with differences in clinical features or treatment success, although this may be due to small sample sizes and the limited use of high-resolution manometry^3,4^. It may also be that the Chicago classification does not appropriately stratify achalasia into meaningful subtypes in CYP. Decisions regarding treatment choice should not necessarily be informed by manometry subtype in CYP until the Chicago classification has been validated in this age group.

Medical treatments did not appear to be effective at reducing symptoms in the present series. The rate of treatment success and time to subsequent intervention were both significantly higher in CYP initially treated with HCM *versus* EBD. However, HCM had a significantly higher rate of complications, including a 9% perforation rate. Similar results have been reported in other studies of CYP with achalasia, with perforations occurring in 3–19% of patients undergoing HCM^5,17,20,21^. Smits *et al.*^3^ observed a higher rate of perforations when HCM was used to treat relapsed disease. In the present study, treatment-naïve patients undergoing HCM did not have fewer complications than those who had undergone EBD before. Similarly, Montalva *et al.*^17^ did not observe an increased risk of oesophageal perforation in patients undergoing HCM who had undergone a previous EBD. However, in the present study, patients undergoing HCM following EBD more frequently required subsequent interventions. This may reflect a more severe disease phenotype, but could also suggest that HCM is less effective after EBD. In contrast, Provenzano *et al.*^21^ reported that previous EBD did not negatively affect HCM outcome in a cohort of 40 CYP. It is likely that first-line HCM is more effective than EBD, but it remains unclear whether HCM is less effective and causes more complications if performed after EBD.

Few studies have reported the long-term outcomes of repeated EBD in CYP with achalasia. In 20–50% of CYP, multiple EBD were required for ongoing symptoms, with 62% subsequently undergoing surgical management^2^. Adult randomized trials have shown that EBD and HCM have comparable rates of treatment success in the short and long term, but repeat EBD is required in 25% of patients^19,22^. In adults with achalasia, younger age at diagnosis is a risk factor for relapse and a requirement for repeat EBD^23^. As such, early HCM may be beneficial in CYP with achalasia to limit the requirement for repeated EBD.

Most surgeons in the present study performed HCM with fundoplication to prevent gastro-oesophageal reflux symptoms after myotomy. The most common choice of fundoplication was a partial anterior wrap. However, there remains no consensus regarding whether fundoplication is necessary, or which type of fundoplication is optimal. A 2022 Cochrane meta-analysis of 571 adult patients with achalasia concluded that the addition of an anterior fundoplication to HCM was not clearly associated with a decrease in acid reflux or an increase in dysphagia symptoms after the surgery^24^. However, total (Nissen) fundoplication did increase the risk of postoperative dysphagia. In the present study, an increased risk of postoperative dysphagia was observed in CYP undergoing HCM with fundoplication. Previous studies in CYP have not identified an association between fundoplication, gastro-oesophageal reflux, and dysphagia^20^. Further work is required to elucidate the relationship between HCM, with and without fundoplication, and long-term outcomes in CYP.

In this study, no POEM procedures were reported and therefore no conclusions can be drawn on the relative efficacy of POEM *versus* HCM and EBD. Multiple reports in the literature^6–9,25–28^ show good short-term outcomes for POEM in adults and CYP, and it is likely that POEM will become part of standard practice in selected high-volume centres with sufficient experience of the technique. A randomized trial comparing POEM to EBD is underway^29^, but studies comparing POEM to HCM, with long-term follow-up, are urgently required.

A strength of this study is that it consists of a large and well described cohort of CYP with achalasia. In contrast, the study is limited by its use of retrospective data, which relies on complete and accurate documentation in the patient record. No diagnostic criteria for achalasia were specified in this study, which may reduce the external validity of the results. Changes in management strategies may have occurred throughout the data collection period, and procedures were not standardized between surgeons or centres. Similarly, at follow-up, the presence of symptoms, gastro-oesophageal reflux disease, dysphagia, and oesophagitis relied on accurate reporting in the patient record, and was based on a mix of endoscopy reports, pH study results and patient reporting. No data on the severity or frequency of symptoms were collected.

Five-year follow-up in this study was limited and, as such, no firm conclusions can be made regarding the long-term outcomes of CYP with achalasia. Due to the challenges of conducting randomized comparative trials in rare paediatric diseases, it is likely that large prospective cohort studies will be required to determine the optimal treatment strategies for achalasia in CYP. A multicentre prospective disease registry with long-term follow-up would provide a data set with sufficient detail to compare treatments and understand the long-term implications of achalasia on the presence, severity and frequency of symptoms, growth, and quality of life. To inform this, core outcomes that are important to healthcare professionals, patients, and their caregivers need to be defined^30^. Similarly, a symptom score that is validated in CYP with achalasia must be created to monitor response to treatment.

There is significant variation in the management of CYP with achalasia in the UK. Due to the low incidence of achalasia in this age group, the authors recommend that CYP should be managed by a multidisciplinary team at experienced centres. HCM appears to be the most effective first-line treatment compared with EBD, although it is associated with a higher complication rate. There is also a suggestion in this data set that HCM performed after EBD is less efficacious. This study reports anticipated outcomes in CYP with achalasia, which include ongoing symptoms in half of all children at the 1-year follow-up. These data will be useful to clinicians, CYP with achalasia, and their caregivers when making decisions about the management of achalasia in this age group.

## Collaborators

The members of the PSTRN Collaborators Group (in alphabetical order) are: Mohamad E. Abdullah (The Royal London Hospital, London, UK); Paul Charlesworth (The Royal London Hospital, London, UK); Liza Y. W. Chong (Royal Hospital for Children and Young People, Edinburgh, UK); Claire Clark (Royal Hospital for Children and Young People, Edinburgh, UK); David Colvin (Royal Belfast Hospital for Sick Children, Belfast, UK); Ahish Desai (The Royal London Hospital, London, UK); Hany Gabra (Great North Children’s Hospital, Newcastle, UK); Stefano Giuliani (Great Ormond Street Hospital, London, UK); Lucinda Tullie (Great Ormond Street Hospital, London, UK); Dale Gracie (Royal Hospital for Children and Young People, Edinburgh, UK); Naotunna Palliyage Nuwanthika Karunaratne (Great Ormond Street Hospital, London, UK); Saravanakumar Paramalingam (Royal Alexandra Children’s Hospital, Brighton, UK); Chiara Pelosi (Birmingham Children’s Hospital, Birmingham, UK); Shazia Perveen (Leicester Children’s Hospital, Leicester, UK); Amulya Saxena (Chelsea Children’s Hospital, London, UK); Giampiero Soccorso (Birmingham Children’s Hospital, Birmingham, UK); Chloe Roy (Great North Children’s Hospital, Newcastle, UK); Iain Yardley (Evelina London Children’s Hospital, London, UK); Dylan Wills (John Radcliffe Hospital, Oxford, UK); Rachel Tan Wei Ying (John Radcliffe Hospital, Oxford, UK).

## Supplementary Material

zraf139_Supplementary_Data

## Data Availability

Data are available upon reasonable request to the authors.

## References

[1] Islam S . Achalasia. Semin Pediatr Surg 2017;26:116–120 28550869 10.1053/j.sempedsurg.2017.02.001

[2] Goneidy J, Cory-Wright J, Zhu L, Malakounides G. Surgical management of esophageal achalasia in pediatrics: a systematic review. Eur J Pediatr Surg 2020;30:13–20 31600801 10.1055/s-0039-1697958

[3] Smits M, van Lennep M, Vrijlandt R, Benninga M, Oors J, Houwen R et al Pediatric achalasia in the Netherlands: incidence, clinical course, and quality of life. J Pediatr 2016;169:110–5.e3 26616251 10.1016/j.jpeds.2015.10.057

[4] Nicolas A, Aumar M, Tran LC, Tiret A, Duclaux-Loras R, Bridoux-Henno L et al Comparison of endoscopic dilatation and Heller’s myotomy for treating esophageal achalasia in children: a multicenter study. J Pediatr 2022;251:134–9.e2 35853483 10.1016/j.jpeds.2022.07.010

[5] Saliakellis E, Thapar N, Roebuck D, Cristofori F, Cross K, Kiely E et al Long-term outcomes of Heller’s myotomy and balloon dilatation in childhood achalasia. Eur J Pediatr 2017;176:899–907 28536813 10.1007/s00431-017-2924-x

[6] Nabi Z, Ramchandani M, Chavan R, Darisetty S, Kalapala R, Shava U et al Outcome of peroral endoscopic myotomy in children with achalasia. Surg Endosc 2019;33:3656–3664 30671667 10.1007/s00464-018-06654-1

[7] Petrosyan M, Mostammand S, Shah AA, Darbari A, Kane TD. Per oral endoscopic myotomy (POEM) for pediatric achalasia: institutional experience and outcomes. J Pediatr Surg 2022;57:728–735 35361482 10.1016/j.jpedsurg.2022.02.017

[8] Shimamura Y, Sato H, Yagi R, Abe H, Shiwaku H, Shiota J et al Clinical characteristics and long-term efficacy of peroral endoscopic myotomy in pediatric achalasia. J Gastroenterol Hepatol 2025;40:1446–1453 40143675 10.1111/jgh.16945

[9] Dimopoulou A, Dimopoulou D, Analitis A, Dimopoulou K, Dellaportas D, Zavras N. Laparoscopic Heller myotomy *versus* peroral endoscopic myotomy in children with esophageal achalasia: a systematic review and meta-analysis. Ann Gastroenterol 2024;37:1–10 39568706 10.20524/aog.2024.0923 PMC11574155

[10] Harris PA, Taylor R, Thielke R, Payne J, Gonzalez N, Conde JG. Research electronic data capture (REDCap)—a metadata-driven methodology and workflow process for providing translational research informatics support. J Biomed Inform 2009;42:377–381 18929686 10.1016/j.jbi.2008.08.010 PMC2700030

[11] Neville JJ, Schaffer S, Eaton S, Hall NJ. Outcome reporting in studies of paediatric achalasia: a systematic review. J Pediatr Gastroenterol Nutr 2025;81:523–529 40544378 10.1002/jpn3.70128 PMC12408982

[12] Delgado-Miguel C, Amarnath RP, Camps JI. Robotic-assisted vs. laparoscopic Heller’s myotomy for achalasia in children. J Pediatr Surg 2024;59:1072–1076 38016851 10.1016/j.jpedsurg.2023.11.003

[13] Wood LSY, Chandler JM, Portelli KE, Taylor JS, Kethman WC, Wall JK. Treating children with achalasia using per-oral endoscopic myotomy (POEM): twenty-one cases in review. J Pediatr Surg 2020;55:1006–1012 32197825 10.1016/j.jpedsurg.2020.02.028

[14] Pachl MJ, Rex D, DeCoppi P, Cross K, Kiely EM, Drake D et al Paediatric laparoscopic Heller’s cardiomyotomy: a single centre series. J Pediatr Surg 2014;49:289–292 24528969 10.1016/j.jpedsurg.2013.11.042

[15] Yadlapati R, Kahrilas PJ, Fox MR, Bredenoord AJ, Prakash Gyawali C, Roman S et al Esophageal motility disorders on high-resolution manometry: Chicago classification version 4.0^©^. Neurogastroenterol Motil 2021;33:e14058 33373111 10.1111/nmo.14058 PMC8034247

[16] Growth Charts - CDC growth charts . https://www.cdc.gov/growthcharts/cdc-growth-charts.htm (accessed September 2025)

[17] Montalva L, Farha E, Hervieux E, Ali L, Rousseau V, Schmitt F et al Complications after Heller myotomy in children: a national multicenter study on the impact of prior endoscopic dilatation and identification of risk factors. Surg Endosc 2024;38:3602–3608 38769183 10.1007/s00464-024-10884-x

[18] Andolfi C, Fisichella PM. Meta-analysis of clinical outcome after treatment for achalasia based on manometric subtypes. Br J Surg 2019;106:332–341 30690706 10.1002/bjs.11049

[19] Moonen A, Annese V, Belmans A, Bredenoord AJ, Bruley des Varannes S, Costantini M et al Long-term results of the European achalasia trial: a multicentre randomised controlled trial comparing pneumatic dilation *versus* laparoscopic Heller myotomy. Gut 2016;65:732 26614104 10.1136/gutjnl-2015-310602

[20] Pacilli M, Davenport M. Results of laparoscopic Heller’s myotomy for achalasia in children: a systematic review of the literature. J Laparoendosc Adv Surg Tech A 2017;27:82–90 27901639 10.1089/lap.2016.0169

[21] Provenzano R, Pulvirenti R, Duci M, Capovilla G, Costantini A, Forattini F et al Laparoscopic Heller–Dor is a persistently effective treatment for achalasia even in pediatric patients: a 25-year experience at a single tertiary center. Eur J Pediatr Surg 2023;33:493–498 36720247 10.1055/s-0043-1760822

[22] Boeckxstaens GE, Annese V, des Varannes SB, Chaussade S, Costantini M, Cuttitta A et al Pneumatic dilation *versus* laparoscopic Heller’s myotomy for idiopathic achalasia. N Engl J Med 2011;364:1807–1816 21561346 10.1056/NEJMoa1010502

[23] Alderliesten J, Conchillo JM, Leeuwenburgh I, Steyerberg EW, Kuipers EJ. Predictors for outcome of failure of balloon dilatation in patients with achalasia. Gut 2011;60:10–16 21068135 10.1136/gut.2010.211409 PMC3002841

[24] Midya S, Ghosh D, Mahmalat MW. Fundoplication in laparoscopic Heller’s cardiomyotomy for achalasia. Cochrane Database Syst Rev 2022;12:CD013386 36478353 10.1002/14651858.CD013386.pub2 PMC9730445

[25] Ponds FA, Fockens P, Lei A, Neuhaus H, Beyna T, Kandler J et al Effect of peroral endoscopic myotomy vs pneumatic dilation on symptom severity and treatment outcomes among treatment-naive patients with achalasia: a randomized clinical trial. JAMA 2019;322:134–144 31287522 10.1001/jama.2019.8859 PMC6618792

[26] van Lennep M, van Wijk MP, Omari TIM, Benninga MA, Singendonk MMJ. Clinical management of pediatric achalasia. Expert Rev Gastroenterol Hepatol 2018;12:391–404 29439587 10.1080/17474124.2018.1441023

[27] Hugova K, Mares J, Hakanson B, Repici A, von Rahden BHA, Bredenoord AJ et al Per-oral endoscopic myotomy *versus* laparoscopic Heller’s myotomy plus Dor fundoplication in patients with idiopathic achalasia: 5-year follow-up of a multicentre, randomised, open-label, non-inferiority trial. Lancet Gastroenterol Hepatol 2025;10:431–441 40112837 10.1016/S2468-1253(25)00012-3

[28] Luvsandagva B, Adyasuren B, Bagachoimbol B, Luuzanbadam G, Bai T, Jalbuu N et al Efficacy and safety of peroral endoscopic myotomy for pediatric achalasia: a nationwide study. Medicine (Baltimore) 2024;103:e38970 39121306 10.1097/MD.0000000000038970 PMC11315545

[29] Mussies C, van Lennep M, van der Lee JH, Singendonk MJ, Benninga MA, Bastiaansen BA et al Protocol for an international multicenter randomized controlled trial assessing treatment success and safety of peroral endoscopic myotomy vs endoscopic balloon dilation for the treatment of achalasia in children. PLoS One 2023;18:e0286880 37796851 10.1371/journal.pone.0286880 PMC10553306

[30] Neville JJ, den Uijl I, Irvine W, Eaton S, Gottrand F, Hall NJ. Development of a core outcome set for paediatric achalasia: a joint ERNICA, ESPGHAN and EUPSA study protocol. BMJ Paediatr Open 2025;9:e003130 10.1136/bmjpo-2024-003130 PMC11831267 39947877

