# Supplementary material for: Management and outcomes of paediatric achalasia: multicentre retrospective study in the UK
Source: BJS Open. 2026 Jan 20;10(1):zraf139. doi: 10.1093/bjsopen/zraf139 (PMC12818012; doi:10.1093/bjsopen/zraf139)
Supplement: zraf139_Supplementary_Data [file zraf139_supplementary_data.docx]

**Management and Outcomes of Paediatric Achalasia: a multicentre retrospective study in the United Kingdom**

Jonathan J Neville^1^*, Esther Westwood^2^, Amanda Ladell^3^, George S Bethell^4^, Rachel Harwood^5^, Nigel J Hall^4^, PSTRN OPERA collaborators

1. Great Ormond Street Institute of Child Health, University College London, London, UK
2. Evelina Children’s Hospital, London, UK
3. Achalasia Action
4. University Surgery Unit, University of Southampton, Southampton, UK
5. Alder Hey Children’s Hospital, Liverpool, UK

**Corresponding author.** Jonathan J Neville, Great Ormond Street Institute of Child Health, 30 Guilford Street, London, WC1N 1EH, Jonathan.neville@nhs.net

**ORCID ID** <https://orcid.org/0000-0002-4049-3896> **Twitter** @Jonny_Neville

**Supplementary Materials - Index**

| **Supplementary Figures and Tables** |  |
| --- | --- |
| Table S1: Clinical features of children and young persons with achalasia at presentation. | *page 2* |
| Table S2: Surgical speciality performing Heller cardiomyotomy (HCM) in children and young persons with achalasia. | *page 2* |
| Table S3: Comparison of outcomes in children and young persons undergoing Heller’s cardiomyotomy (HCM) with and without a fundoplication. | *page 2* |
| Figure S1: Z-score for weight at diagnosis and one-year follow-up. | *page 3* |
|  |  |

**Supplementary Figures and Tables**

Table S1: Clinical features of children and young persons with achalasia at presentation.

| **Clinical Feature** | **Frequency (n = 126)** |
| --- | --- |
| Dysphagia | 93 (74%) |
| Vomiting | 67 (53%) |
| Weight loss | 49 (39%) |
| Regurgitation | 42 (33%) |
| Chest pain | 19 (15%) |
| Reflux | 16 (13%) |
| Nocturnal cough | 12 (10%) |
| Choking | 8 (6%) |
| Failure to gain weight | 7 (6%) |
| Nausea | 6 (5%) |
| Abdominal pain | 5 (4%) |
| Recurrent respiratory tract infections | 3 (2%) |
| Slow eating, eating with excessive fluids or bolus impaction | 3 (2%) |
| Other | 4 (3%) |

Table S2: Surgical speciality performing Heller cardiomyotomy (HCM) in children and young persons with achalasia. Operating surgeon speciality was unspecified in one case.

|  | **Paediatric surgeons**  **(n = 89)** | **Adult surgeons**  **(n = 2)** | **Combined adult and paediatric surgeons**  **(n = 6)** | **p-value** |
| --- | --- | --- | --- | --- |
| Any complications | 14 (16%) | 1 (50%) | 1 (17%) | 0.434 |
| Re-do HCM | 7 (8%) | 0 (0%) | 0 (0%) | 0.712 |

Table S3: Comparison of outcomes in children and young persons undergoing Heller’s cardiomyotomy (HCM) with and without a fundoplication.

|  | **HCM without fundoplication**  **(n = 32)** | **HCM with fundoplication**  **(n = 66)** | **p-value** |
| --- | --- | --- | --- |
| Any complications | 2 (6%) | 12 (18%) | 0.134 |
| Treatment success | 13 (41%) | 39 (59%) | 0.086 |
| Re-do HCM | 4 (13%) | 3 (5%) | 0.310 |
| Post-HCM GORD at one-year | 3 (9%) | 5 (8%) | 0.714 |
| Post-HCM dysphagia at one-year | 4 (13%) | 21 (32%) | 0.049* |

*Significant result. GORD – gastro-oesophageal reflux disease.


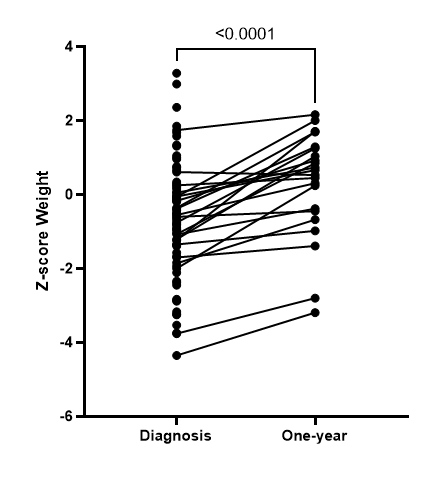


Figure S1: Z-score for weight at diagnosis (n = 105) and one-year follow-up (n = 22).
